# Supplementary material for: Bilayer Kagome Borophene with Multiple van Hove Singularities
Source: Adv Sci (Weinh). 2023 Oct 15;11(37):2305059. doi: 10.1002/advs.202305059 (PMC11462296; doi:10.1002/advs.202305059)
Supplement: Supplementary file 1 — Supporting Information [file ADVS-11-2305059-s001.pdf]

ADVANCED SCIENCE
Open Access

Supporting Information

for *Adv. Sci.*, DOI 10.1002/advs.202305059

Bilayer Kagome Borophene with Multiple van Hove Singularities

Qian Gao, Qimin Yan, Zhenpeng Hu and Lan Chen*

Supporting Information

Bilayer Kagome Borophene with Multiple van Hove Singularities

Qian Gao, Qimin Yan, Zhenpeng Hu, Lan Chen*

Qian Gao, Zhenpeng Hu
School of Physics, Nankai University, Tianjin 300071, China
E-mail: zphu@nankai.edu.cn

Qimin Yan
Department of Physics, Northeastern University, Boston, MA 02115, USA

Lan Chen
Institute of Physics, Chinese Academy of Sciences, Beijing 100190, China

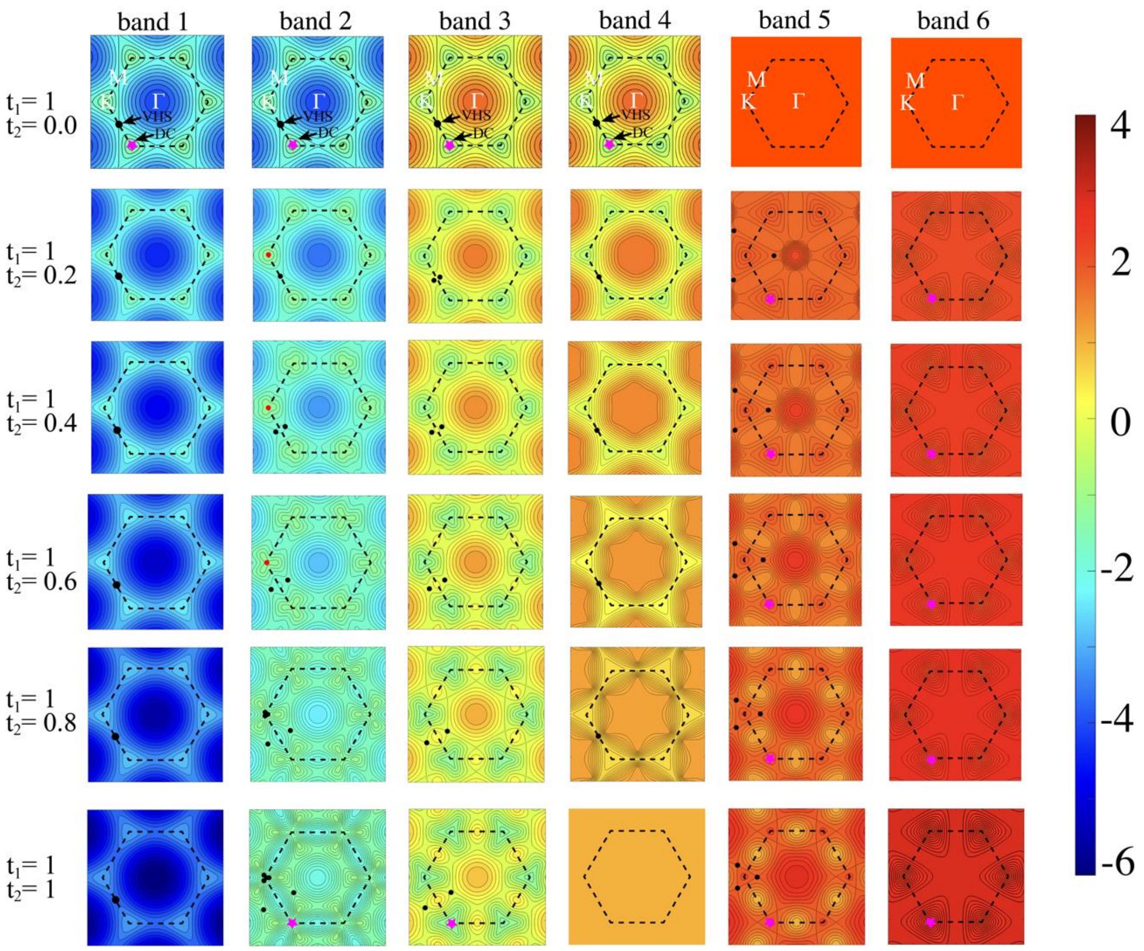

Figure S1. Energy contours of specific bands with hopping amplitudes $t_1 = 1$, and t_2 varying from 0 to 1. The black and red circles represent the sites of VHS and HOVHS, and the magenta pentagram represents the site of DC (Dirac cone).

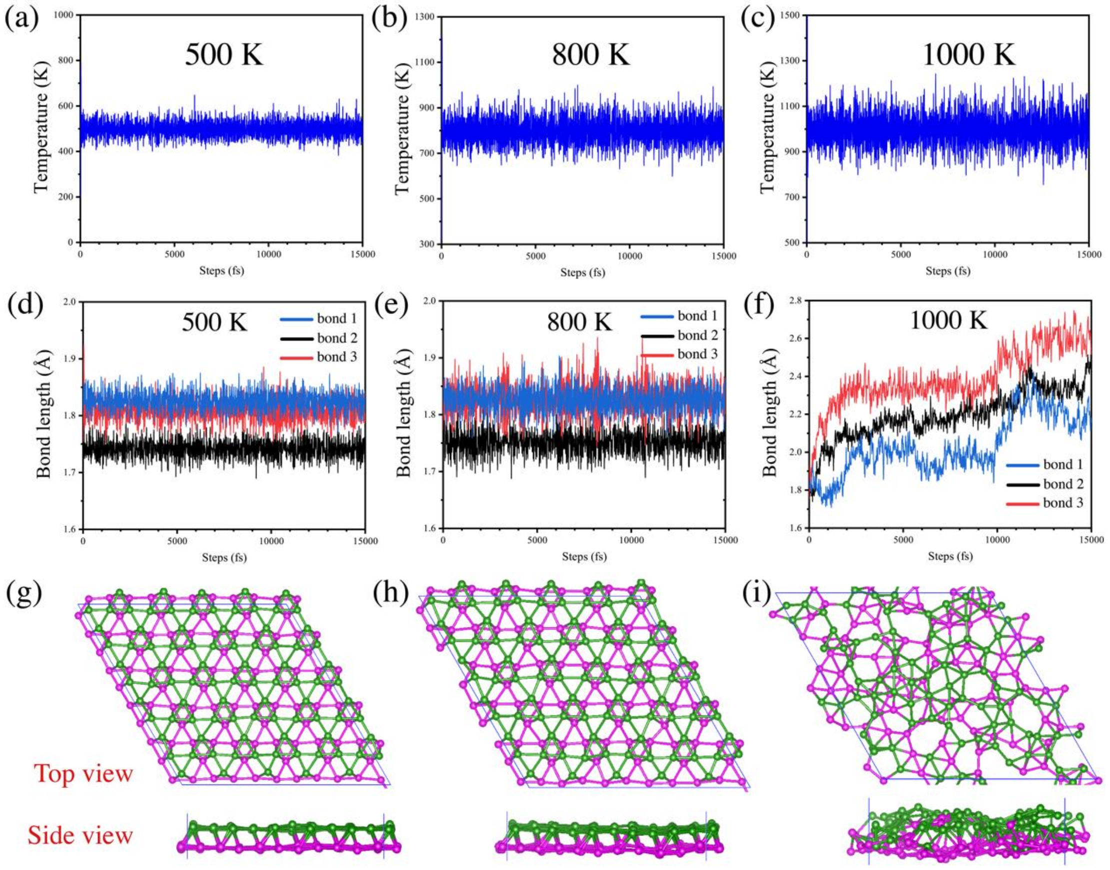

Figure S2. Molecular dynamic simulations at T= (a) 500, (b) 800, (c) 1000 K, and (d-f) the changes of three bonding types, as well as (g-i) the corresponding structures at the final state. The green and magenta colors separately represent the top and bottom atoms at the original state, respectively.

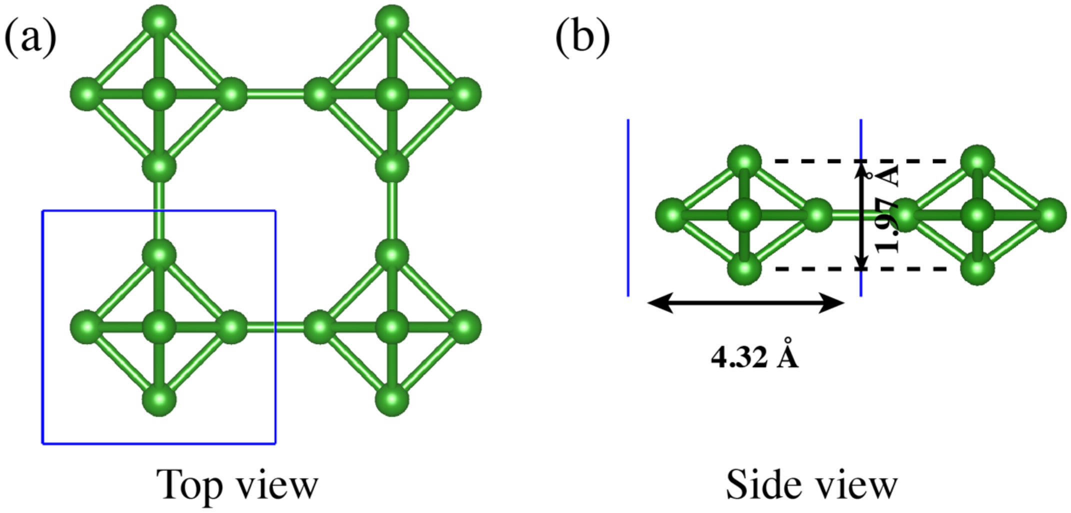

Figure S3. (a) Top and (b) side views of 2D-B_6 monolayer. The lattice constant of 4.32 Å is consistent with the data in Ref. [1].

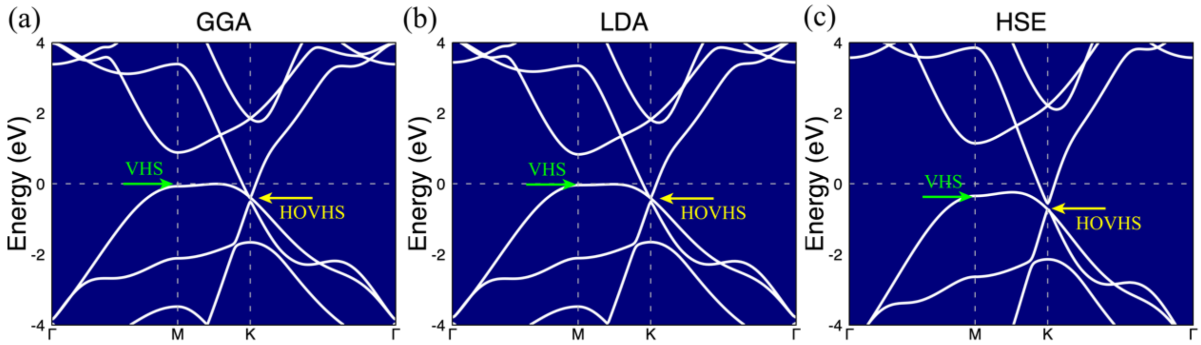

Figure S4. Band structures of BK-borophene along symmetry lines of the Brillouin zone based on (a) GGA-PBE, (b) LDA, and (c) HSE methods.

Table S1. The energy positions (E_{VHS} and E_{HOVHS}) of conventional and high order van Hove singularities near the Fermi level, and the Fermi velocity (v_F) of KB-borophene under the different DFT functionals.

Properties	*GGA(PBE)*	*LDA*	*HSE*
E_{VHS} *(eV)*	-0.065	-0.037	-0.346
E_{HOVHS} *(eV)*	-0.385	-0.412	-0.730
v_F *(m/s)*	1.34×10^6	1.31×10^6	1.63×10^6

Reference:

[1] N.V. Tkachenko, D. Steglenko, N. Fedik, N.M. Boldyreva, R.M. Minyaev, V.I. Minkin, A.I. Boldyrev, Phys. Chem. Chem. Phys., 21 (2019) 19764-19771.
